# Microbial-enzymatic coupling drives nitrogen stabilization during static pile composting of rice straw amended with contrasting nitrogen sources

**DOI:** 10.3389/fmicb.2026.1839803

**Published:** 2026-06-19

**Authors:** Qingran Guo, Jiawei Li, Yutao Peng, Jing Li, Hongzhao Li, Xiao Chen, Baige Zhang

**Affiliations:** 1 Key Laboratory for New Technology Research of Vegetable, Vegetable Research Institute, Guangdong Academy of Agricultural Sciences, Guangzhou, China; 2 School of Agriculture and Biotechnology, Sun Yat-sen University, Shenzhen, China

**Keywords:** enzyme activity, microbial community, nitrogen transformation, organic amendments, straw composting

## Abstract

Nitrogen loss during straw composting substantially undermines nutrient recycling efficiency and the agronomic value of finished compost products. Clarifying how different nitrogen sources mediate nitrogen transformation and nitrogen retention in organic fractions is therefore essential for improving compost maturity and nitrogen retention. This study evaluated how three nitrogen-rich amendments with divergent chemical characteristics (chicken manure, fish meal, and soybean powder) influenced nitrogen transformation, organic nitrogen fractionation, enzyme dynamics, and microbial succession during static pile composting of rice straw. Compost maturity varied significantly among treatments (*P* < 0.05), with germination indices of 100% for soybean powder, 90.1% for fish meal, and 84.9% for chicken manure. Chicken manure primarily promoted a mineralization-oriented nitrogen transformation pattern characterized by rapid ammonium accumulation and subsequent nitrification, accompanied by elevated urease activity and enrichment of Firmicutes. In contrast, fish meal and soybean powder were associated with nitrogen transformation patterns involving stronger proteolytic and oxidative enzyme activities and greater nitrogen retention in relatively stable organic fractions, resulting in significant increases in amine nitrogen and hydrolysable unknown nitrogen (HUN), a relatively stable organic nitrogen fraction, by 45.1–139% relative to the control (*P* < 0.05). Coordinated proteolytic and oxidative enzyme activities, together with enrichment of *Thermobifida*, Actinobacteriota, and *Aspergillus*, were strongly associated with HUN formation. Overall, protein-rich nitrogen sources were more conducive to microbial-enzymatic interactions associated with organic nitrogen stabilization, whereas chicken manure favored nitrogen mineralization. These findings demonstrate that nitrogen sources can shape nitrogen transformation toward greater inorganic nitrogen production or higher organic nitrogen retention, thereby providing practical insights for improving nitrogen retention and compost quality in straw composting systems.

## 1 Introduction

With billions of tons of crop residues, predominantly rice straw, produced globally each year, prevailing disposal practices such as open-field dumping and incineration continue to cause severe environmental pollution and considerable resource losses (Bai et al., 2023; Zhang et al., 2025). Developing cost-effective treatment and recycling technologies is therefore essential for advancing sustainable agricultural systems. Composting represents a promising eco-friendly approach to organic waste management, integrating straw with other biodegradable materials to accelerate decomposition processes (Pan et al., 2024; Wang et al., 2025). The compost obtained serves as a valuable organic fertilizer that enhances soil fertility and crop productivity (Wei et al., 2022; Oueld Lhaj et al., 2025). Nevertheless, the high lignocellulosic recalcitrance of straw significantly slows biodegradation and extends the overall composting period (El Fels et al., 2024). Moreover, its characteristically high carbon-to-nitrogen ratio (often exceeding 40) necessitates supplementation with nitrogen-rich materials to achieve the optimal range of 25–30 for efficient microbial activity (Zhang W. et al., 2022). Consequently, optimizing the initial C/N ratio is fundamental to improving compost quality and shortening the composting period.

During composting, the initial C/N ratio plays a pivotal role in regulating organic matter decomposition and nitrogen transformations. Organic nitrogen is first mineralized to ammonium nitrogen (NH_4_^+^-N). This ammonium can then be assimilated by microorganisms, oxidized to nitrate nitrogen (NO_3_^–^-N) through nitrification, or volatilized as ammonia under high-temperature and alkaline conditions (Baiano et al., 2021). In addition, subsequent nitrification-denitrification can convert reactive nitrogen into N_2_O and N_2_, leading to further gaseous nitrogen losses (Stieglmeier et al., 2014; Rose et al., 2020). These nitrogen transformations are largely mediated by microbial communities and their associated enzymes, which regulate lignocellulose degradation, organic nitrogen depolymerization, ammonification, and humification during composting (El Fels et al., 2024). Enzyme-mediated depolymerization releases small nitrogen-containing compounds that can either enter mineralization pathways or be incorporated into more stable organic nitrogen pools during humification (Zhou et al., 2018; Ren et al., 2020). In this study, microbial-enzymatic coupling refers to the coordinated interaction between microbial community succession and extracellular enzyme activities that jointly regulate the partitioning of nitrogen into mineralized versus relatively recalcitrant organic forms during composting (Ren et al., 2020; El Fels et al., 2024). Consequently, the selection of nitrogen sources influences not only the initial C/N ratio, but also microbial succession, enzyme activities, and the partitioning of nitrogen between mineralization and organic nitrogen retention. This highlights the importance of understanding biologically mediated nitrogen transformation processes to improve straw decomposition efficiency and nitrogen retention.

Nitrogen sources are important drivers of microbial community assembly and compost quality (Zhu et al., 2021; Zhao et al., 2025a). Among the commonly used nitrogen-rich amendments, chicken manure (CM), fish meal (FM), and soybean powder (SP) represent three distinct types of organic nitrogen amendments: fecal-based, animal-derived protein-based, and plant-derived protein-based materials, respectively. Studies have shown that CM promotes thermophilic bacterial growth during maize straw composting, thereby accelerating organic matter degradation (Wang Y. et al., 2024). CM amendment has also been associated with greater microbial biomass and Bacillus enrichment during the thermophilic phase, contributing to rapid lignocellulose decomposition and nitrate accumulation (Zhou Z. et al., 2024). FM is rich in protein and long-chain unsaturated fatty acids that improve nutrient availability and microbial metabolic efficiency (Rimoldi et al., 2021). Previous work has shown that FM amendment increases total hydrolysable nitrogen (THN) and is associated with enhanced Thermobifida activity during thermophilic and maturation phases (Zhou and Yao, 2020). Because protein degradation generates amino compounds that can be further transformed into more stable organic nitrogen fractions during humification, FM may favor nitrogen retention in organic fractions rather than rapid mineral nitrogen accumulation (Zhou et al., 2018; Sun et al., 2024). In addition, its sulfur-containing amino acids may support microbial glutathione synthesis and enhance resistance to oxidative stress during high-temperature composting (Zhao et al., 2022). In contrast, the fiber and lipid components of SP can influence organic matter decomposition and promote humification during composting (Qi et al., 2022). SP-treated compost has been reported to show substantially higher polyphenol oxidase activity, facilitating lignin degradation and humic acid formation (Zhou J. et al., 2024). However, the efficacy of these nitrogen-rich amendments depends strongly on the chemical characteristics of the composting substrate. While previous research has explored their individual roles in various organic wastes, their comparative impacts on nitrogen transformation and stabilization during rice straw composting under identical initial C/N conditions remain unclear.

Microorganisms play a pivotal role in driving the composting process (Sánchez et al., 2017; Papale et al., 2021), exerting strong influences on both compost quality and process dynamics (Chandna et al., 2013). Distinct microbial taxa exhibit specific preferences for different nitrogen sources (Roca-Mesa et al., 2020; Ivanova et al., 2021). Numerous studies have shown that selecting appropriate nitrogen amendments can stimulate beneficial and efficient microbial consortia, thereby improving compost performance and final quality (Ma et al., 2025). For instance, varying the proportions of soybean powder and sugarcane bagasse in vermicomposting significantly enhanced the germination index (GI) of pakchoi and radish (Cai et al., 2022). Likewise, supplementing chicken manure compost with corn straw reshaped the dominant microbial community and increased the abundance of thermophilic taxa (Zhang et al., 2018), whereas wheat straw composting did not induce notable shifts in microbial community structure (Zhang et al., 2016; Zhao et al., 2025b). Despite growing interest in optimizing nitrogen-rich amendments for straw composting, several critical gaps remain. Most studies have focused primarily on total and inorganic nitrogen dynamics, potentially overlooking the redistribution of organic nitrogen into fractions of varying stability. Consequently, the extent of nitrogen retention driven by true stabilization of organic nitrogen, as opposed to the temporary accumulation of inorganic forms, remains unclear. Additionally, while microbial succession and enzyme activities are recognized as pivotal to composting processes, their coordinated responses to differing nitrogen-source chemistries under uniform initial C/N conditions have yet to be systematically assessed. Furthermore, static aerated pile systems are known for providing controlled aeration and reducing nitrogen loss potential (Tong et al., 2019; Sun et al., 2022), yet the specific nitrogen transformation patterns across distinct organic nitrogen sources within these systems are not well-characterized.

To address these gaps, this study investigated the effects of three representative nitrogen-rich amendments, namely chicken manure, soybean powder, and fish meal, during the static pile composting of rice straw under a uniform initial C/N ratio. We systematically quantified compost maturity and physicochemical changes, characterized inorganic nitrogen and hydrolysable organic nitrogen fractions, measured key enzyme activities related to nitrogen and organic matter transformation, and analyzed how these factors correlated with bacterial and fungal community succession. In this study, amendment-dependent nitrogen transformation was further interpreted as two contrasting patterns: a mineralization-oriented pattern, reflected by greater conversion of organic nitrogen into inorganic forms, and a retention/stabilization-oriented pattern, reflected by greater partitioning of nitrogen into hydrolysable organic fractions during composting. We hypothesized that these distinct patterns would be associated with coordinated shifts in microbial communities and extracellular enzyme activities driven by nitrogen-source chemistry. By integrating these datasets, we aimed to elucidate whether different nitrogen-source chemistries were associated with distinct nitrogen transformation patterns and differences in nitrogen retention among relatively recalcitrant organic fractions. The findings provide process-level evidence to support selecting nitrogen-rich amendments rationally, ultimately accelerating compost maturity and enhancing organic nitrogen retention.

## 2 Materials and methods

### 2.1 Composting materials and experimental design

This study was conducted over 35 days, from 1 October to 4 November 2023, in a greenhouse located in Baini Town, Sanshui District, Guangdong Province, China (23.27°N, 112.91°E). Four treatments were established, consisting of the control (CK) and three nitrogen-rich amendments: chicken manure (CM), soybean powder (SP), and fish meal (FM). Detailed information on the composting formulations for each treatment is provided in Supplementary Table 1, and all treatments were performed in triplicate. Rice straw collected from a local farm was air-dried and cut into 3–5 cm lengths. The key physicochemical properties of all raw materials are provided in Supplementary Table 2.

### 2.2 Aerobic composting set-up

The composting system employed in this study is shown in Supplementary Figure 1. Each treatment received microbial inoculants at a rate of 5 mL kg^–1^ (dry basis), together with the designated nitrogen source and a portion of mature compost to ensure sufficient microbial activation and nutrient availability. Continuous aeration was maintained at 0.7 L min^–1^ kg^–1^ total solids. This rate was selected based on previous composting studies to ensure adequate oxygen supply for aerobic decomposition while minimizing excessive heat and moisture loss (Gao et al., 2010). The mixture of rice straw and nitrogen-rich amendments was adjusted to an initial C/N ratio of 25, which is widely considered optimal for microbial activity and composting efficiency (Guo et al., 2012). Composting materials were manually mixed at regular intervals to maintain substrate homogeneity without disrupting the static aeration regime. Samples were collected every 4 days during the first 2 weeks and every 7 days thereafter until day 35. At each sampling point, 40 g of well-mixed compost was collected and divided into two equal portions. One portion was stored at 4 °C for physicochemical analyses, and the other was preserved at −20°C for microbial and molecular assessments.

### 2.3 Compost sample collection and analysis

Representative samples were collected from each composting pile to monitor the C/N ratio, pH, moisture content (MC), and temperature. For pH and electrical conductivity (EC) measurements, a 1:10 (w:v) compost-to-water suspension was shaken for 30 min and analyzed using a pH meter (Hanna HI 221 Microprocessor, Italy) and a conductivity probe (WTW LF 320, Germany), respectively. Total carbon (TC) and total nitrogen (TN) were determined with an elemental analyzer (vario EL III, CHNOS Elemental Analyzer, Elementar, Germany), and the C/N ratio was calculated accordingly. Key enzyme activities, including urease, polyphenol oxidase (PPO), dehydrogenase (DHA), catalase (CAT), and protease (ALPT), were quantified using commercial assay kits (Grace Biotechnology, China) according to the manufacturer’s protocols.

The concentrations of total hydrolysable nitrogen (THN), amino acid nitrogen (AAN), amino sugar nitrogen (ASN), amine nitrogen (AN), and hydrolysable unknown nitrogen (HUN) were determined using the acid hydrolysis method (Chen, 2013). Organic nitrogen fractions were extracted from the hydrolysate prepared by refluxing compost samples with 6 mol L^–1^ HCl (Keeney and Bremner, 1964). After acid hydrolysis, the contents of AAN, ASN, AN, and HUN were analyzed. Briefly, 1 g of compost sample, 0.1 mL of octanol, and 50 mL of 6 mol L^–1^ HCl were added to 100 mL digestion tubes, which were then sealed and incubated in a digestion furnace at 105 °C for 12 h. The four organic nitrogen fractions were subsequently measured using the Kjeldahl method with appropriate additives (Bremner and Keeney, 1966), and calculated according to Equations 1, 2. Specifically, THN was determined via Kjeldahl digestion with H_2_SO_4_, AN was measured in the hydrolysate using MgO, and the combined AN and ASN content was assessed using a phosphate-borate buffer. AAN was quantified by reacting the hydrolysate with NaOH, citric acid, and ninhydrin in a water bath at 100 °C, followed by Kjeldahl analysis.


ASN=(AN+ASN)-AN (1)

HUN=THN-(AN+ASN+AAN) (2)

After evenly distributing the samples on filter paper, 10 seeds were incubated in the dark at 20 °C for 48 h. Seed germination and root length were recorded to evaluate the effects of each treatment. The germination index (GI) was calculated using the following equation (Equation. 3) (Xie et al., 2022):


$$\text{GI}\left(\%\right)=\frac{\mathrm{Seed}\mathrm{germination}\left(\%\right)\times \mathrm{root}\mathrm{length}\mathrm{of}\mathrm{treatment}}{\mathrm{Seed}\mathrm{germination}\left(\%\right)\times \mathrm{root}\mathrm{length}\mathrm{of}\mathrm{control}}\times 100$$ (3)

### 2.4 DNA extraction and 16S rRNA genes sequencing

Compost samples were submitted to Shanghai Meiji Bio-medical Technology Co., Ltd. for high-throughput sequencing. Microbial DNA was extracted using a FastDNA Spin Kit for soil (MPBio), and DNA purity was assessed with a NanoDrop 2000 UV-Vis spectrophotometer (Thermo Scientific, Wilmington, United States). The V3-V4 hypervariable region of the bacterial 16S rRNA gene was amplified using primers 338F (ACTCCTGGGAGGCGAGCGAG) and 806R (GGACTACHVGGTWTCTAAT), while the fungal ITS2 region was amplified using barcoded primers ITS7 and ITS4.

### 2.5 Statistical analysis

All experiments were conducted in triplicate, and results are presented as mean ± standard deviation. Statistical analyses were performed using IBM SPSS Statistics 25.0. One-way analysis of variance (ANOVA) followed by Duncan’s multiple range test (*P* < 0.05) was used to assess significant differences among treatments. High-throughput sequencing data for bacterial 16S rRNA and fungal ITS2 amplicons were processed on the Majorbio Cloud Platform (Majorbio Bio-Pharm Technology Co., Ltd., Shanghai, China).

## 3 Results and discussion

### 3.1 Shifts of Physicochemical parameters in composting systems

**FIGURE 1:**
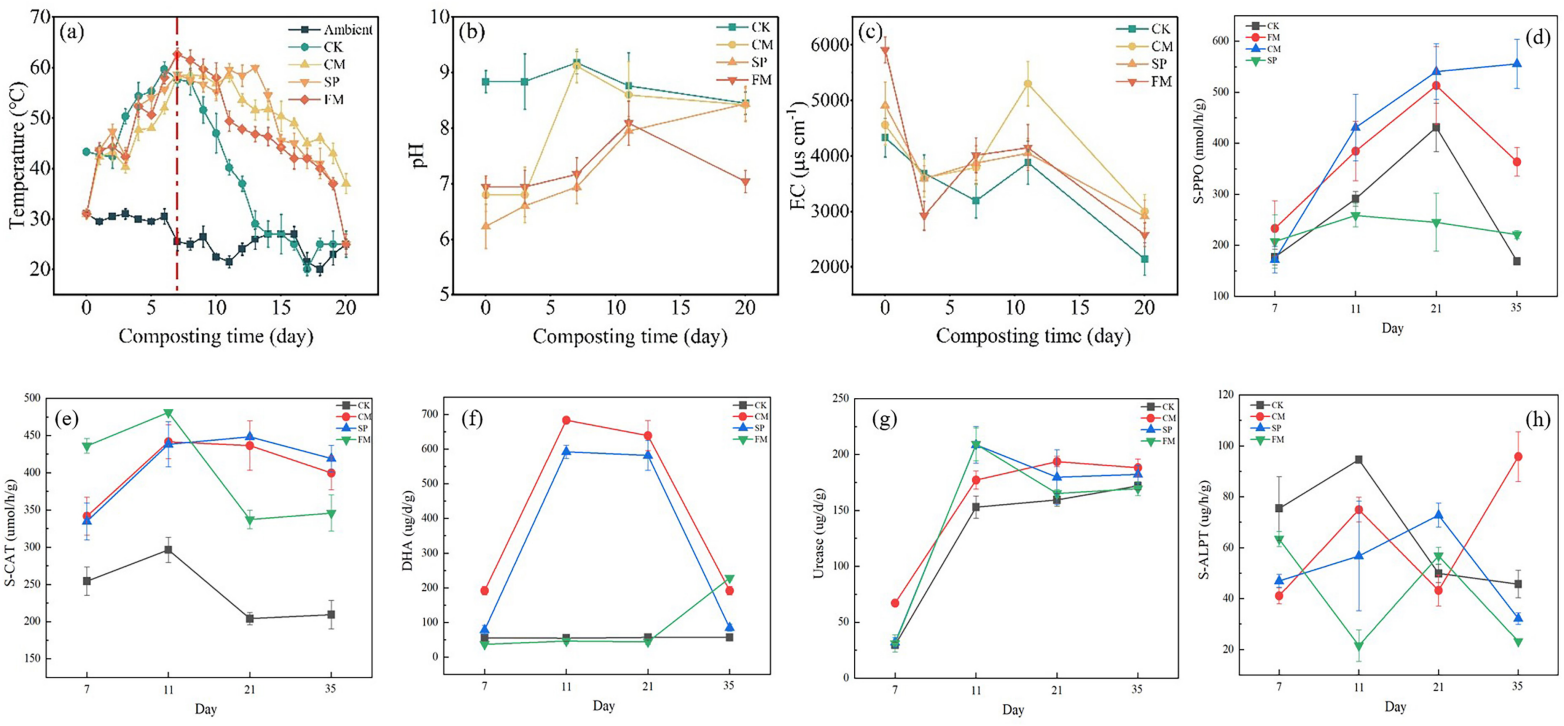
Changes in physicochemical properties and enzyme activities during composting. Bulk temperature **(a)**, pH **(b)**, and electrical conductivity (EC) **(c)**. Activities of polyphenol oxidase **(d)**, catalase **(e)**, dehydrogenase **(f)**, urease **(g)**, and protease **(h)**. CM, chicken manure; SP, soy powder; FM, fish meal.

After day 35 of composting, the germination index (GI) was used to assess whether nitrogen-rich amendments could accelerate compost maturity. The GI results clearly differentiated the treatments, with SP achieving the highest maturity (100%), followed by FM (90.1%), CM (84.9%), and CK (80.8%) (Table 1). These results indicate that the type of nitrogen amendment significantly influenced compost maturity. To further elucidate the internal factors driving these differences, correlation analysis and structural equation modeling (SEM) were performed to quantify the relative contributions of abiotic variables to GI formation (Supplementary Figures 2, 3). Organic matter (OM) degradation emerged as the primary direct determinant of GI, whereas pH and temperature mainly acted as indirect regulators by affecting OM transformation. Thermophilic duration varied among treatments. CK, FM, CM, and SP maintained temperatures above 55 °C for 4, 5, 6, and 8 days, respectively, and FM reached the highest peak temperature (60 °C) (Figure 1a). OM content exhibited corresponding treatment-dependent trends, with SP retaining the highest residual OM, followed by CM and FM. These observations align with the GI results, suggesting that SP provided conditions most conducive to stable OM transformation and enhanced compost maturity.

**TABLE 1:** Maturity indices of different compost treatments on day 35.

Treatment	CK	CM	SP	FM
BD (g cm^–3^)	0.53 ± 0.01b	0.77 ± 0.01a	0.54 ± 0.01b	0.73 ± 0.04a
GI (%)	80.8 ± 1.6d	84.9 ± 3.3c	100 ± 9.4a	90.1 ± 7.1b
OM (g kg^–1^)	41.6 ± 0.28c	57.0 ± 0.15b	69.2 ± 0.63a	56.9 ± 0.76b
TN (g kg^–1^)	1.34 ± 0.01c	2.04 ± 0.01a	1.93 ± 0.02b	1.94 ± 0.03b
TP (g kg^–1^)	0.47 ± 0.01c	1.93 ± 0.01a	0.84 ± 0.01b	0.83 ± 0.01b
TK (g kg^–1^)	3.11 ± 0.02a	3.23 ± 0.03a	3.19 ± 0.03a	3.23 ± 0.01a
NO_3_^–^-N (mg kg^–1^)	7.38 ± 0.04a	6.81 ± 0.03b	1.85 ± 0.08c	1.84 ± 0.13c
NH_4_^+^-N (mg kg^–1^)	10.8 ± 0.02a	6.76 ± 0.05b	5.52 ± 0.20c	3.89 ± 0.12d
Total nutrients (%)	4.91 ± 0.03c	7.20 ± 0.02a	5.95 ± 0.02b	6.00 ± 0.02b

Different lowercase letters within a column indicate significant differences among treatments at *P* < 0.05. BD, bulk density; GI, germination index; OM, organic matter; TN, total nitrogen; TP, total phosphorus; TK, total potassium; NO_3_^–^-N, nitrate nitrogen; NH_4_^+^-N, ammonium nitrogen. CK, control; CM, chicken manure; SP, soy powder; FM, fish meal.

Further analysis showed that SP’s superior performance was closely associated with its exceptionally high plant-protein content, which accounted for approximately 73% of its organic constituents (Supplementary Figure 4). The abundance of proteinaceous substrates provided sustained carbon and nitrogen sources, supporting continuous microbial metabolism and facilitating efficient OM transformation (Chang et al., 2019). In addition, SP maintained a near-neutral initial pH (average 7.1), which helped minimize ammonia volatilization and provided a more stable metabolic environment during composting (Figure 1b; Zhou Z. et al., 2024). In the CM treatment, the extended thermophilic phase was associated with its relatively higher NH_4_^+^-N concentration compared with SP and FM. CM showed an NH_4_^+^-N content that was 18.3–42.5% higher than that in SP and FM (*P* < 0.05) (Table 1). This pattern is consistent with the rapid hydrolysis of urea and urate compounds in chicken manure, which generated additional ammonium during early thermophilic conditions. In addition, the alkaline environment in CM, with pH values exceeding 8.0 during peak thermophilic stages, may have imposed physiological constraints on microbial activity, thereby reducing the efficiency of OM mineralization (Zhou and Yao, 2020). In contrast, FM exhibited a comparatively shorter thermophilic phase, which may be partly related to its high content of sulfur-containing amino acids, particularly methionine. Consistent with its high amino acid content (∼65%, Supplementary Figure 4b), such compounds could potentially influence microbial thermal tolerance and community stability, ultimately leading to earlier cessation of high-temperature activity (Wang et al., 2022).

Bulk density (BD) further contributed to explaining the differences in GI among treatments. SEM analysis revealed a negative effect of BD on GI (β = −0.17) (Supplementary Figure 3b). According to established standards (Schaub-Szabo and Leonard, 1999), composts with BD values below 300 kg m^–3^ are unsuitable for agricultural use. All final products in this study exceeded this threshold (Table 1), indicating that their structural quality was acceptable. Previous research has identified 0.50–0.55 g cm^–3^ as the optimal BD range for balancing aeration and microbial activity (Chang et al., 2019). Excessively high BD can limit oxygen diffusion to aerobic microorganisms (Amuah et al., 2022), which may account for the relatively lower GI observed in certain treatments. The intermediate BD of the SP treatment (0.54 g cm^–3^) likely facilitated adequate aeration and supported Actinobacteria-driven OM degradation, thereby contributing to its superior compost maturity (Zhao et al., 2016; Table 1; Supplementary Figure 3b).

Although this duration is shorter than the typical 30–60 days required for straw-based composting (Malik et al., 2021), the combined optimization of OM degradation, pH stability, thermophilic persistence, and BD likely enabled all treatments to reach GI values above 80% within 35 days. This rapid stabilization is largely attributable to the patented static aerobic reactor (Chinese Patent^®^ ZL 202311416205.3), which improves oxygen diffusion, enhances thermal retention, and supports microbial colonization, thereby accelerating composting without compromising product quality.

### 3.2 Transformation and redistribution of nitrogen fractions

The above results indicate that nitrogen availability strongly regulates OM degradation and compost maturity, highlighting nitrogen mineralization and retention in organic fractions as central drivers of the differences observed among treatments. Given this, clarifying how nitrogen transforms during composting is essential for understanding the mechanisms behind these treatment-specific effects. Accordingly, we further analyzed the dynamics of different nitrogen fractions. Because nitrogen speciation directly shapes microbial metabolism, OM turnover, and humification processes, quantifying both inorganic and organic nitrogen forms provides critical insight into how nitrogen-rich amendments influence compost development and functional outcomes.

Across treatments, CM exhibited substantially higher concentrations of both NO_3_^–^-N and NH_4_^+^-N than the other treatments, whereas FM showed the lowest NH_4_^+^-N content (Table 1). In composting systems, organic nitrogen is typically mineralized to NH_4_^+^-N and subsequently oxidized to NO_3_^–^-N, with NO_3_^–^-N often accumulating during the maturation phase as nitrification persists while ammonification slows (Sánchez-Monedero et al., 2001). Consistent with this pattern, the elevated NH_4_^+^-N and NO_3_^–^-N observed in CM reflect rapid nitrogen mineralization associated with poultry manure inputs (Zhu et al., 2021). This trend is attributable to the hydrolysis of uric acid and urea, which generated abundant NH_4_^+^ under thermophilic aerobic conditions, followed by partial nitrification. By contrast, FM exhibited suppressed NO_3_^+^-N accumulation despite achieving a high thermophilic peak and substantial organic matter degradation. This decoupling between OM decomposition and nitrification suggests that nitrogen transformation in FM was not dominated by inorganic accumulation. Such inhibition of nitrification has been reported in substrates rich in sulfur-containing amino acids, which may alter microbial activity and redirect nitrogen away from nitrate formation (Rose et al., 2020; Zhang et al., 2024).

Beyond inorganic nitrogen, the amendments substantially altered the distribution and transformation of organic nitrogen fractions (Table 2). Organic nitrogen was partitioned into AAN, ASN, AN, and HUN. Among these, AAN, ASN, and AN represent relatively labile components and are typically classified as bioavailable organic nitrogen (BON). In contrast, HUN is generally considered a relatively recalcitrant hydrolysable organic nitrogen fraction and is often discussed in relation to nitrogen retention in more stable organic forms during composting (Chen, 2013; Sun et al., 2024). All nitrogen-rich amendments significantly expanded the organic nitrogen pools compared with CK (*P* < 0.05), with FM yielding the highest THN content (12.4 g kg^–1^). During the thermophilic phase, rapid degradation of proteinaceous substrates triggered pronounced rises in AN. Compared with CK, AN increased by 576, 373 and 330% in FM, CM, and SP, respectively (*P* < 0.05) (Zhou et al., 2018). Although ASN remained relatively low in absolute abundance, it increased by 131% in CM and 268% in FM, likely due to sufficient energy derived from easily degradable organics that supported microbial growth and biosynthesis (Ren et al., 2020). The inclusion of straw in SP further optimized pile structure and aeration, promoting microbial colonization and metabolic activity (Chen, 2013). AAN, which is closely linked to microbial biomass turnover and glucose-bound nitrogen, accumulated through both microbial assimilation and the release of intracellular nitrogen following cell lysis during community succession (Zhou et al., 2018).

**TABLE 2:** Contents of organic nitrogen fractions under different compost treatments.

Treatment	THN	AN	ASN	AAN	HUN
CK	3.03 ± 0.33d	0.37 ± 0.03c	0.19 ± 0.03c	0.77 ± 0.09d	1.33 ± 0.15c
CM	9.80 ± 0.57b	1.75 ± 0.11b	0.44 ± 0.08b	2.66 ± 0.17b	3.19 ± 0.16a
SP	7.23 ± 0.33c	1.59 ± 0.06b	0.23 ± 0.03c	1.89 ± 0.09c	1.93 ± 0.07b
FM	12.4 ± 0.66a	2.50 ± 0.12a	0.70 ± 0.06a	3.57 ± 0.34a	3.10 ± 0.16a

Different lowercase letters within a column indicate significant differences among treatments at *P* < 0.05. THN, total hydrolysable nitrogen; AN, amine nitrogen; ASN, amino sugar nitrogen; AAN, amino acid nitrogen; HUN, hydrolysable unknown nitrogen. CK, control; CM, chicken manure; SP, soy powder; FM, fish meal.

Notably, HUN increased by 45.1–139% in CM, SP, and FM relative to CK (*P* < 0.05), suggesting greater retention of nitrogen in relatively stable organic forms during composting. Although both FM and SP promoted HUN accumulation, their nitrogen transformation patterns differed from CM, which was characterized primarily by inorganic nitrogen enrichment. FM produced the highest concentrations of THN, AN, ASN, AAN, and HUN, followed by CM and SP, confirming that nitrogen-rich inputs, particularly FM, substantially enhanced organic nitrogen pools. Taken together, these contrasting inorganic and organic nitrogen profiles indicate two distinct nitrogen transformation tendencies: CM was associated with more rapid mineralization and inorganic nitrogen accumulation, whereas FM and SP were associated with greater nitrogen retention in organic fractions, especially HUN. This divergence suggests that nitrogen fate was regulated not only by substrate availability but also by amendment-specific microbial processing tendencies. To quantify the relative contributions of these fractions to NH_4_^+^-N formation, we constructed a structural equation model (SEM) (Figure 4d). The results showed that HUN exhibited the strongest positive association with NH_4_^+^-N, followed by ASN and AAN, whereas AN played only a minor role. These relationships indicate that stabilized organic nitrogen pools remained closely linked to mineral nitrogen during composting. However, because humic structures were not directly characterized using spectroscopic techniques (e.g., FTIR or ^13^C NMR), the association between HUN accumulation and humification should be interpreted as indirect evidence. Future studies integrating molecular-level analyses are needed to verify whether these nitrogen fraction shifts correspond to structural incorporation into humified matrices.

### 3.3 Comparison of enzyme activity among treatments

Since shifts in nitrogen forms arise from microbially mediated processes, understanding the enzymatic mechanisms that drive these transformations is critical. Enzymes serve as direct biochemical indicators of nitrogen mineralization, organic matter decomposition, and microbial functional dynamics during composting. Given that nitrogen-rich amendments substantially altered both inorganic and organic nitrogen pools (Tables 1, 2), evaluating the activities of key nitrogen- and carbon-transforming enzymes provides mechanistic insight into how these amendments influenced nitrogen transformation during composting. Accordingly, we quantified the temporal dynamics of urease, polyphenol oxidase (PPO), dehydrogenase (DHA), catalase (CAT), and protease (ALPT) throughout the composting process (Figure 1).

Temporal variations in enzyme activities during composting are presented in Figure 1. Urease activity was initially low in all treatments but increased sharply during the thermophilic phase. It peaked on day 11 and subsequently declined toward maturity (Figure 1g). This trajectory is consistent with rapid ammonification and organic nitrogen mineralization during the early composting stages (Zhang et al., 2025). On day 11, FM showed the highest urease activity. By day 35, however, CM exhibited the highest urease activity among the treatments, suggesting a greater persistence of ammonification-related enzymatic activity under this amendment. PPO activity remained relatively stable during the heating phase (Figure 1d). However, by day 35, CM exhibited the highest PPO activity, representing a 228% increase relative to CK (*P* < 0.05). SP maintained consistently moderate PPO activity throughout the composting period.

DHA also varied substantially among treatments (Figure 1f). On day 11, DHA in CM and SP increased by 1,153 and 986% (*P* < 0.05), respectively, relative to CK, indicating intensified microbial oxidative metabolism during peak decomposition (Ding et al., 2025). DHA subsequently declined across all treatments during the maturation stage, consistent with reduced microbial respiration as labile substrates diminished and compost stability improved (Campos et al., 2019). CAT activity increased in all treatments upon entering the thermophilic phase (Figure 1e). Specifically, CAT rose from 254.3, 341.0, 334.0, and 436.0 mmol h^–1^g^–1^ in CK, CM, SP, and FM to 296.0, 441.0, 438.0, and 481.0 mmol h^–1^ g^–1^ (*P* < 0.05), respectively. The increase in CAT activity likely reflects an adaptive microbial antioxidant response to elevated oxidative stress during the thermophilic stage, when intensified respiration and rapid organic matter oxidation can increase the accumulation of reactive oxygen species such as H_2_O_2_. The higher CAT activity in nitrogen-amended piles further suggests that enhanced substrate availability stimulated microbial metabolism and thereby increased the need for reactive oxygen detoxification (Wang et al., 2021). ALPT activity generally declined toward the end of composting, with the exception of CM (Figure 1h). The comparatively low protease activity observed in CK likely reflects the limited availability of nitrogen-rich and proteinaceous substrates, which reduced both microbial demand for proteolytic decomposition and the induction of extracellular protease synthesis (Magotra et al., 2022). Notably, FM and SP exhibited concurrent elevations in protease, PPO, and DHA during the active composting phase. This pattern indicates coordinated hydrolytic and oxidative enzyme responses under these amendments. This enzyme pattern is consistent with a sequential processing pattern in FM and SP, whereby proteinaceous substrates were first hydrolyzed into soluble peptides and amino acids through protease activity, followed by oxidative transformation mediated by PPO and microbial redox metabolism reflected by DHA. Such coordinated enzyme activity has been associated with the transformation and retention of reactive nitrogenous compounds in organic forms (Wang J. et al., 2024; Zhou Z. et al., 2024). Consistent with this interpretation, FM and SP also showed greater accumulation of HUN, suggesting greater retention of nitrogen in organic forms during composting, although the present data do not allow direct attribution of this pattern to specific enzyme activities. In contrast, by day 35, CM exhibited the highest urease activity together with elevated inorganic nitrogen levels, suggesting a more mineralization-oriented nitrogen transformation pattern under this amendment.

Overall, the incorporation of diverse organic nitrogen sources significantly stimulated microbial enzyme production (de Mendonça Costa et al., 2016; Qiu et al., 2021), but the specific chemical nature of these substrates influenced the predominant patterns of nitrogen transformation. FM and SP were associated with coordinated proteolytic and oxidative enzyme activity and greater retention of nitrogen in relatively stable organic forms, whereas CM was associated with higher urease activity and greater inorganic nitrogen accumulation. These results help explain the divergent nitrogen fraction patterns observed among treatments.

### 3.4 Structure of bacterial and fungal diversity and correlation analysis

**FIGURE 2:**
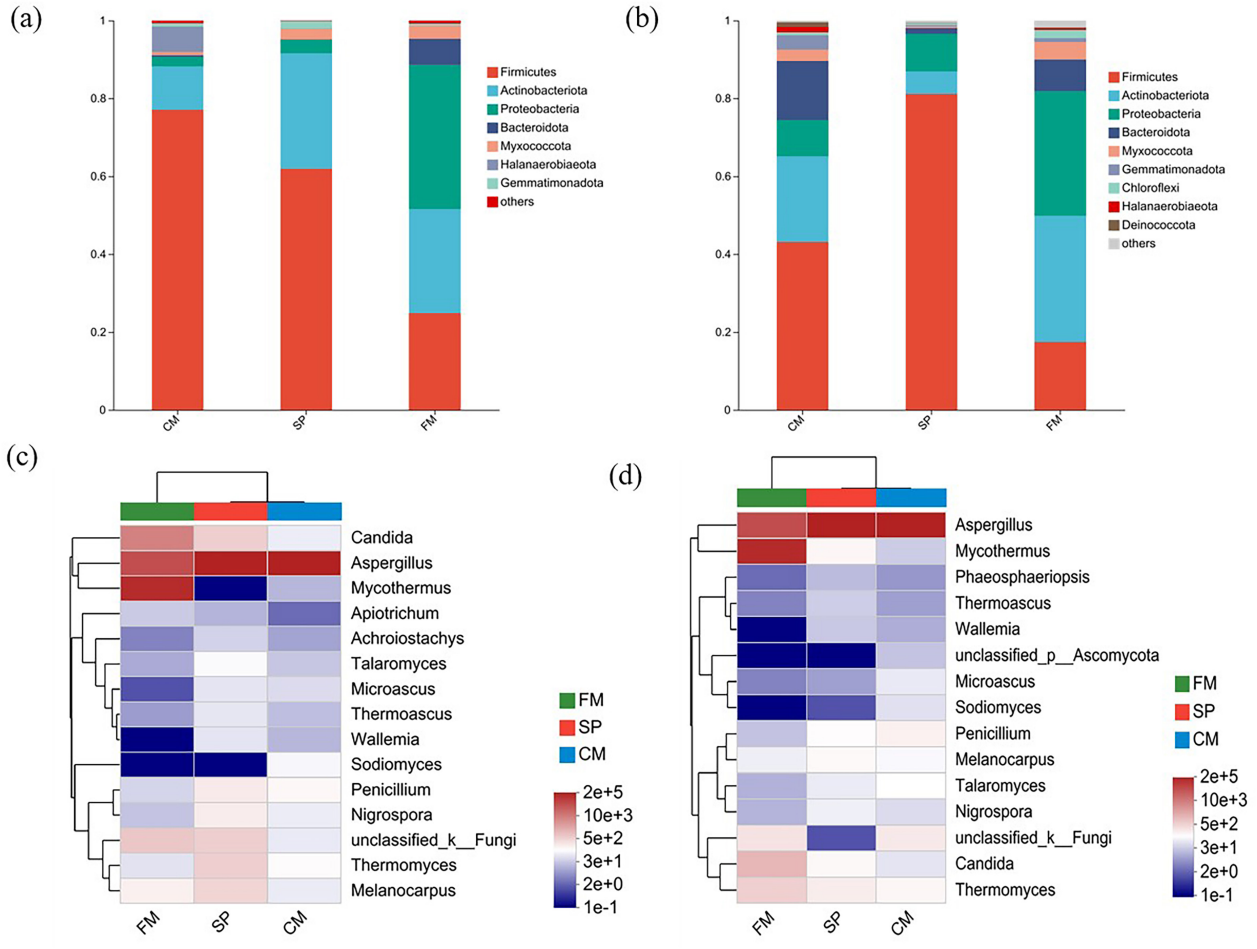
Microbial community composition during composting. Relative abundances of bacterial communities at the phylum level on day 12 **(a)** and day 20 **(b)**. Heat maps of the top 15 fungal genera on day 12 **(c)** and day 20 **(d)**. CM, chicken manure; SP, soy powder; FM, fish meal.

As the activities of nitrogen- and carbon-transforming enzymes are fundamentally dependent on the composition of the compost microbial community, it is necessary to examine how nitrogen amendments restructure microbial assemblages. Therefore, we further analyzed bacterial and fungal community dynamics to determine how nitrogen sources shaped microbial succession and how these changes corresponded to composting performance. At the phylum level, bacterial communities exhibited pronounced, treatment-specific shifts throughout the process (Figures 2a,b). Across all piles, *Firmicutes*, *Proteobacteria*, and *Actinobacteriota* were the dominant phyla, together comprising the majority of 16S rRNA gene reads. However, their relative abundances varied substantially with nitrogen source, suggesting that different amendments selected for distinct microbial assemblages. At the genus level, Thermobifida was notably enriched in FM and SP. Fungal community analysis also showed enrichment of Aspergillus under these treatments, indicating potential differences in oxidative transformation processes among amendments. *Firmicutes* were most abundant in CM (77.1%), *Actinobacteriota* were enriched in SP (29.6%), and *Proteobacteria* reached their highest proportion in FM (37.1%) (*P* < 0.05). These differences likely reflect amendment-specific substrate characteristics, which selected for distinct bacterial groups (Xie et al., 2022). The dominance of Firmicutes in CM is consistent with previous studies showing that this phylum is highly competitive during thermophilic composting and actively participates in rapid organic matter degradation (Chandna et al., 2013; Wang et al., 2022). In contrast, the enrichment of *Actinobacteriota* in SP may be associated with their contribution to the degradation of more recalcitrant lignocellulosic substrates, whereas the higher proportion of Proteobacteria in FM suggests adaptation to amendment-specific nutrient conditions (Zhao et al., 2016; Zhang et al., 2018). On day 20, bacterial communities showed marked restructuring. In SP, *Firmicutes* increased to 81.2% (*P* < 0.05), whereas their proportion declined in CM. Differential abundance analysis revealed significant enrichment of *Bacteroidota* in FM on day 12 (*P* < 0.05) (Supplementary Figure 5b), while *Firmicutes* and *Actinobacteriota* dominated all treatments on day 20 (Supplementary Figure 5d). Circos plots further illustrated distinct community configurations across amendments (Supplementary Figures 5e,f). *Firmicutes* consistently dominated during the thermophilic phase, consistent with their well-documented capacity for rapid organic matter degradation and contributions to NH_4_^+^-N accumulation through proteolytic and cellulolytic activities (Li et al., 2025). The decline of *Firmicutes* in CM may reflect the depletion of easily degradable carbon substrates, as carbon limitation is known to constrain the growth of thermophilic *Firmicutes* (Bai et al., 2023).

**FIGURE 3:**
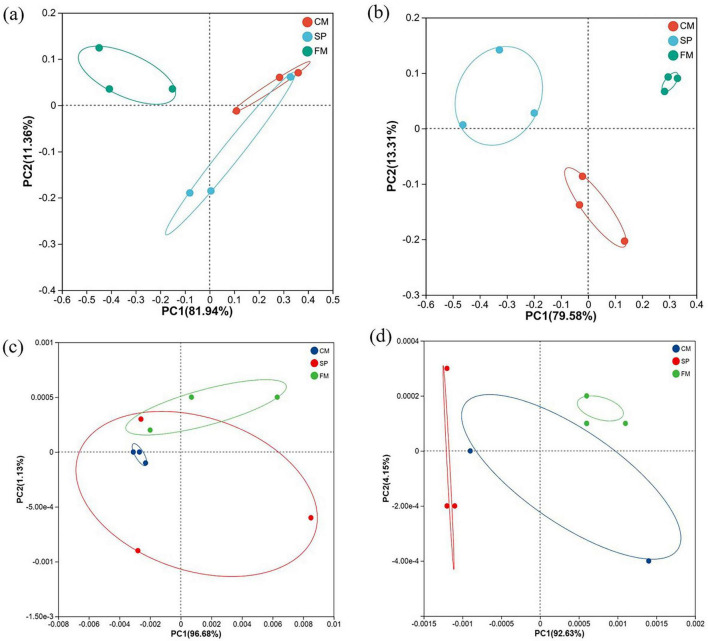
Principal coordinate analysis (PCoA) of microbial communities during composting. PCoA of bacterial communities on day 12 **(a)** and day 20 **(b)**, and fungal communities on day 12 **(c)** and day 20 **(d)**. CM, chicken manure; SP, soy powder; FM, fish meal.

At the genus level, *Thermobifida* dominated FM and SP on day 12, whereas *Bacillus* was most abundant in CM (Supplementary Figure 5a). On day 20, shifts in dominance were evident: SP became dominated by *Bacillus*, while *Thermobifida* increased in CM (Supplementary Figure 5c). *Bacillus* species are well-adapted to high-temperature environments and participate in lignocellulose degradation through diverse extracellular enzymes (Gharechahi et al., 2023). The increased proportion of *Thermobifida* in CM may reflect its ability to degrade recalcitrant substrates once easily decomposable carbon is depleted. Such shifts suggest that nitrogen amendments shaped substrate availability, which in turn influenced microbial successional trajectories. Fungal communities also showed treatment-specific patterns. The dominant fungal genera throughout composting were *Aspergillus*, *Candida*, and *Mycothermus* (Figures 2c,d). *Candida* was more abundant during the early stage (day 12), whereas *Aspergillus* predominated at maturity. *Mycothermus* was particularly enriched in FM, aligning with previous findings that this genus thrives at high temperatures and contributes to the degradation of polymeric and carboxylic compounds (Wang et al., 2018a,b). PCoA demonstrated clear temporal separation of microbial communities between day 12 and day 20, indicating substantial temporal shifts under different nitrogen amendment treatments (Figure 3). *Eurotiomycetes* remained a dominant fungal class under all treatments, consistent with their roles in decomposing lignocellulosic substrates and contributing to compost maturation and pathogen suppression (Wu et al., 2022). Overall, these results demonstrate that nitrogen-rich amendments strongly influenced the succession of bacterial and fungal populations, driving distinct community shifts that paralleled differences in organic matter degradation and nitrogen transformation patterns across treatments.

### 3.5 Relationships between environmental factors, microbial structure, and nitrogen fractions

**FIGURE 4:**
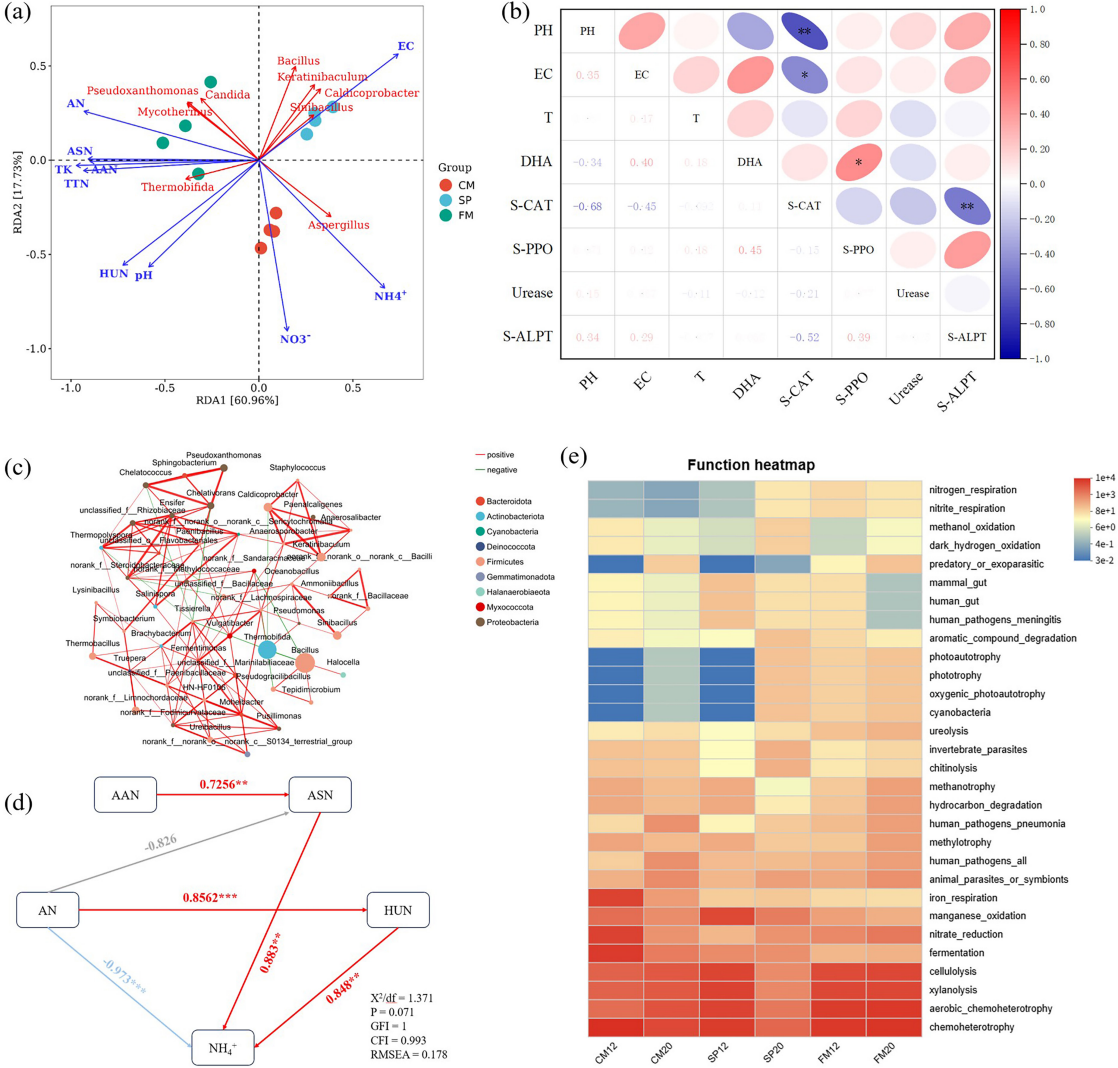
Relationships among compost properties, microbial communities, enzyme activities, and nitrogen transformation during composting. Redundancy analysis of physicochemical properties and microbial genera **(a)**. Correlation analysis of enzyme activities and compost parameters on days 12 and 15 **(b)**. Co-occurrence network analysis of bacterial genera under CM, FM, and SP treatments on days 12 and 20 **(c)**. Transformation of nitrogen fractions during composting **(d)**. Predicted microbial functional composition based on FAPROTAX under different treatments on days 12 and 20 **(e)**. CM, chicken manure; SP, soy powder; FM, fish meal.

Microbial succession is conventionally recognized as the central driver of nutrient turnover during composting, where heterotrophic decomposers facilitate the depolymerization of organic macromolecules to support microbial metabolism (Zhou Z. et al., 2024). Although nitrogen amendments clearly altered microbial community structures, the extent to which these shifts aligned with changes in nitrogen forms and environmental conditions required further clarification. To disentangle these relationships and identify the biochemical mechanisms underlying nitrogen transformation, redundancy analysis (RDA) was performed. This analysis evaluated associations between environmental variables, nitrogen fractions, and microbial community composition (Figure 4a). The first two RDA axes explained 78.7% of the total variation, indicating that environmental variables and nitrogen speciation were primary drivers of microbial community assembly. Among the nitrogen fractions, AN, AAN, and NH_4_^+^-N showed strong positive correlations with *Thermobifida*, *Pseudoxanthomonas*, *Candida*, and *Mycothermus*, suggesting that these taxa may be associated with protein depolymerization and early-stage nitrogen mineralization. These microorganisms are known to secrete thermostable proteases and dehydrogenases, facilitating the conversion of macromolecular proteins into amino acids and smaller nitrogenous compounds (Dong et al., 2020; Morales-Borrell et al., 2020). In contrast, *Bacillus*, *Caldicoprobacter*, and *Keratinibaculum* displayed closer associations with NO_3_^–^-N and HUN, indicating possible associations with downstream nitrogen transformation and retention in relatively stable organic fractions during compost maturation (Ren et al., 2020; Lin et al., 2025). Notably, HUN clustered closely with PPO and DHA activities in FM and SP treatments (Figure 4b). This pattern suggests that the formation of relatively stable organic nitrogen fractions was coupled with oxidative enzymatic processes rather than representing residual undecomposed nitrogen. This pattern is consistent with a possible sequential transformation pathway in which protein substrates are initially hydrolyzed into amino acid nitrogen, followed by oxidative processing that facilitates incorporation of nitrogen into more stable organic pools. Structural equation modeling (SEM) further clarified these relationships (Figure 4d). Organic matter degradation exerted a strong direct effect on compost maturity, while nitrogen fractions influenced NH_4_^+^-N formation primarily through HUN, ASN, and AAN. These results indicate that stabilized organic nitrogen pools remained dynamically linked to mineral nitrogen, highlighting continuous exchange between organic and inorganic nitrogen forms during composting.

Distinct amendment-specific nitrogen transformation patterns were evident. In CM, nitrogen was primarily present as uric acid and urea, which are rapidly hydrolyzed to NH_4_^+^-N (Tawfik et al., 2023). This accounts for the elevated NH_4_^+^-N concentrations and enhanced urease activity observed during the thermophilic phase, reflecting swift mineralization of poultry-derived nitrogen. By contrast, nitrogen in FM and SP is largely bound in high-molecular-weight proteins, amino acids, and lipids (Pelyuntha et al., 2022; Zhang S. et al., 2022), requiring enhanced proteolytic activity for depolymerization. This biochemical constraint favored microbial strategies oriented toward organic nitrogen processing rather than rapid mineralization, consistent with higher AAN, ASN, and HUN accumulation in FM and SP. Collectively, these results demonstrate that nitrogen source chemistry imposed distinct selection pressures on microbial communities and enzymatic pathways, thereby directing nitrogen fate toward either mineralization-dominated turnover (CM) or enzyme-mediated retention in relatively stable organic fractions (FM and SP). These contrasting patterns should be interpreted as relative and amendment-dependent rather than mutually exclusive. Inorganic nitrogen production and organic nitrogen retention occurred simultaneously across all treatments, but to different extents.

### 3.6 Network analysis of core microbial communities and nitrogen fractions

Building on the above findings regarding relationships between nitrogen dynamics and microbial communities, we further examined genus-level co-occurrence patterns for days 12 and day 20 across the CM, FM, and SP treatments (Figure 4c). The resulting network comprised 47 nodes and 88 edges, indicating a structured pattern of taxon–taxon associations that varied with amendment type. *Firmicutes* and *Proteobacteria* were the dominant phyla represented in the network. Within these groups, Bacillus showed relatively high network centrality, particularly in CM, with multiple associations with other genera. Given the known ability of some Bacillus species to produce urease and protease (Tiwari et al., 2019), this pattern may be consistent with the higher NH_4_^+^-N concentrations and urease activity observed in CM. However, these network links represent statistical co-occurrence relationships and should not be interpreted as direct ecological interactions, cooperation, or causal control over nitrogen transformation. In contrast, FM and SP networks showed relatively greater connectivity involving *Thermobifida* and *Pseudoxanthomonas*, which were associated with AAN and ASN. These taxa are known producers of thermostable proteases and oxidative enzymes, facilitating degradation of complex proteinaceous substrates under thermophilic conditions (Stieglmeier et al., 2014; Gharechahi et al., 2023). Their prominence in the network, together with the higher PPO and DHA activities observed in FM and SP, suggests that these treatments were associated with microbial communities linked to organic nitrogen depolymerization and transformation rather than predominantly inorganic nitrogen accumulation. Nevertheless, positive associations in the network may also reflect shared environmental preferences or similar responses to substrate conditions, rather than direct biological interactions. Overall, the network patterns were broadly consistent with the amendment-dependent differences observed in nitrogen fractions and enzyme activities. CM was associated with a more centralized network structure together with higher inorganic nitrogen accumulation, whereas FM and SP were associated with more distributed co-occurrence patterns and greater retention of nitrogen in organic fractions, including HUN. These results suggest that nitrogen source chemistry was associated with distinct microbial assembly patterns during composting, although direct functional interactions and mechanistic pathways cannot be confirmed by co-occurrence analysis alone.

### 3.7 Predicted microbial functions under different composting treatments

To further examine potential functional differences among treatments, FAPROTAX analysis was applied to infer potential biogeochemical functions based on the 16S rRNA gene dataset. The inferred profiles showed strong enrichment of chemoheterotrophy, aerobic chemoheterotrophy, cellulolysis, xylanolysis, and nitrogen respiration-related functions across the CM, FM, and SP treatments (Figure 4e). These functions may contribute broadly to nutrient cycling, organic-matter turnover, and microbial energy metabolism (Zhou et al., 2023).

Despite these shared functional groups, nitrogen-related pathways differed substantially among treatments. In CM, ureolysis, nitrate reduction, and fermentation-related functions were strongly enriched. This pattern suggests an increased potential for rapid hydrolysis of uric acid and urea into NH_4_^+^, together with enhanced nitrogen turnover under fluctuating oxygen conditions (Islam et al., 2021). This predicted functional enrichment was consistent with the elevated inorganic nitrogen contents observed in CM and suggests a greater potential for rapid nitrogen turnover, which may increase the risk of nitrogen loss under this amendment (Zheng et al., 2024).

In contrast, the FM and SP treatments were more strongly associated with cellulolysis and xylanolysis, indicating that their microbial communities may have allocated relatively greater metabolic potential toward lignocellulose degradation (Oh et al., 2017). This predicted functional orientation was consistent with the observed increases in amino acid nitrogen and amino sugar nitrogen in FM and SP, as well as the greater accumulation of HUN. Together, these results suggest greater retention of nitrogen in organic fractions in these systems. Taken together, these predicted functional differences were consistent with the broader treatment-dependent patterns observed in nitrogen transformation, with CM showing stronger association with inorganic nitrogen accumulation and FM and SP showing stronger association with organic nitrogen retention (Dong et al., 2025).

**FIGURE 5:**
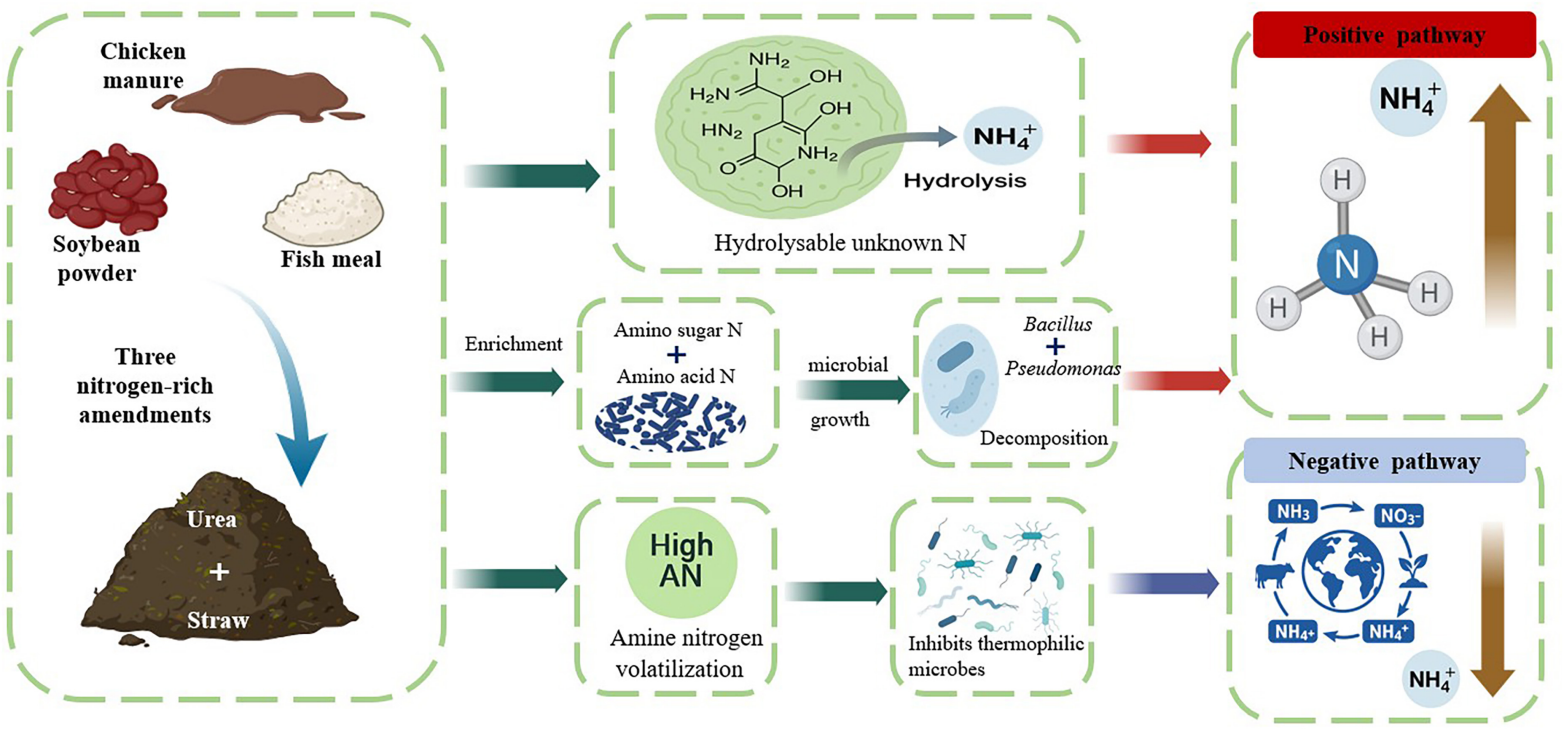
Conceptual diagram illustrating the potential positive and negative mechanisms regulating ammonium nitrogen bioavailability during composting.

Mechanistically, these inferred functional profiles suggest that different nitrogen sources may be associated with distinct nitrogen transformation patterns (Figure 5). CM was linked to more rapid and potentially loss-prone nitrogen turnover, whereas FM and SP were linked to comparatively slower but more organic nitrogen-retentive patterns. It is important to note, however, that FAPROTAX predictions are inference-based and rely on taxonomic identity rather than direct gene detection. Therefore, these results should be interpreted as putative functional potential rather than direct evidence of actual microbial activity. More broadly, the relationships identified here through FAPROTAX annotation and co-occurrence analysis provide inferential support but do not constitute direct functional validation. Future investigations integrating metagenomic or metatranscriptomic approaches, functional gene quantification, isotope tracing, or direct process-level measurements are needed to verify the abundance, expression, and activity of pathways involved in ammonification, nitrification, denitrification, and organic nitrogen stabilization.

## 4 Conclusion

This study demonstrates that the chemical nature of nitrogen sources, rather than merely the initial C/N ratio, plays a central role in shaping nitrogen transformation patterns during rice straw composting. Specifically, chicken manure was associated with a mineralization-dominated pattern characterized by rapid ammonification, subsequent nitrification, and inorganic nitrogen accumulation. By contrast, fish meal and soybean powder promoted greater retention of reactive nitrogen within relatively stable organic fractions, particularly HUN. Such divergent nitrogen transformation patterns were tightly linked to distinct enzymatic profiles and microbial interactions, ultimately influencing nitrogen retention and compost maturity. Practically, these findings highlight that selecting appropriate nitrogen-rich amendments can help regulate nitrogen fate throughout straw composting. Complex protein-based nitrogen sources such as fish meal and soybean powder may therefore be more effective than chicken manure when the objective is to enhancing organic nitrogen stabilization, mitigating nitrogen loss risks, and accelerating compost maturity. Future studies should validate the field-scale agronomic performance of these HUN-enriched composts with a focus on nitrogen release kinetics, crop nitrogen utilization, and subsequent improvements in soil nitrogen fertility.

## Data Availability

The original contributions presented in this study are included in this article/Supplementary material, further inquiries can be directed to the corresponding author.
