# Supplementary material for: Microbial-enzymatic coupling drives nitrogen stabilization during static pile composting of rice straw amended with contrasting nitrogen sources
Source: Front Microbiol. 2026 Jun 19;17:1839803. doi: 10.3389/fmicb.2026.1839803 (PMC13328361; doi:10.3389/fmicb.2026.1839803)
Supplement: Supplementary file 1 [file Data_Sheet_1.docx]

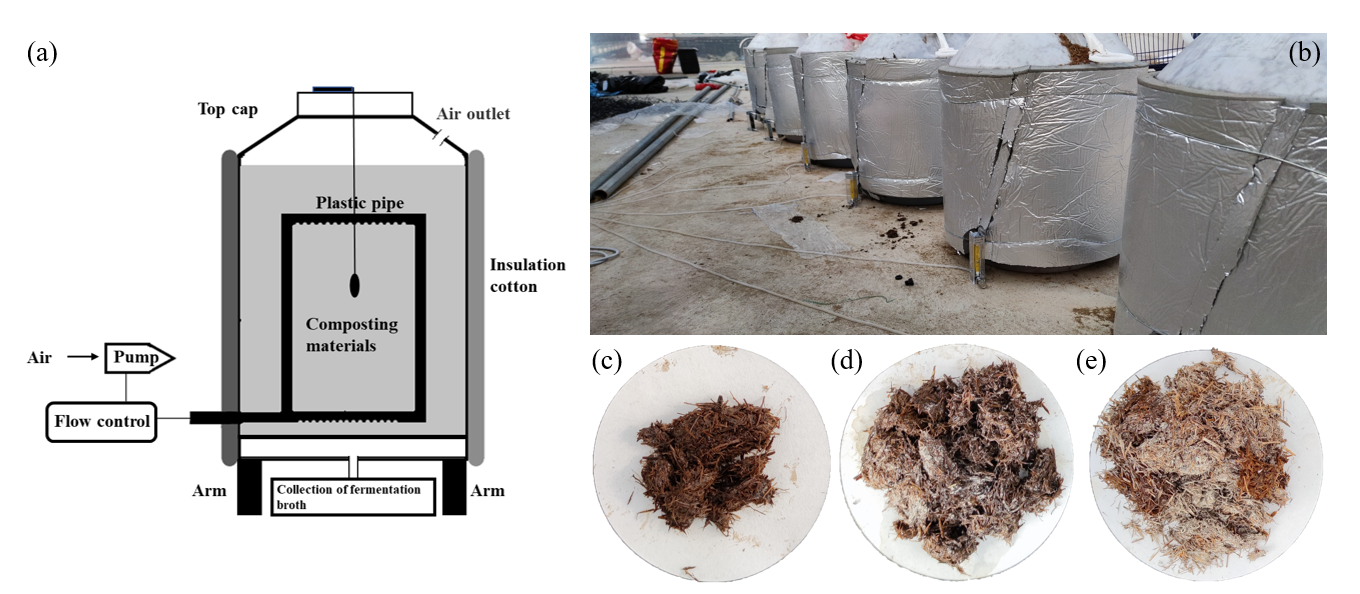


**Figure S1**. Composting system and products: (a) schematic diagram of straw composting device, (b) physical reactor, (c-e) final compost products from chicken manure (CM), fish meal (FM), and soybean powder (SP) treatments.


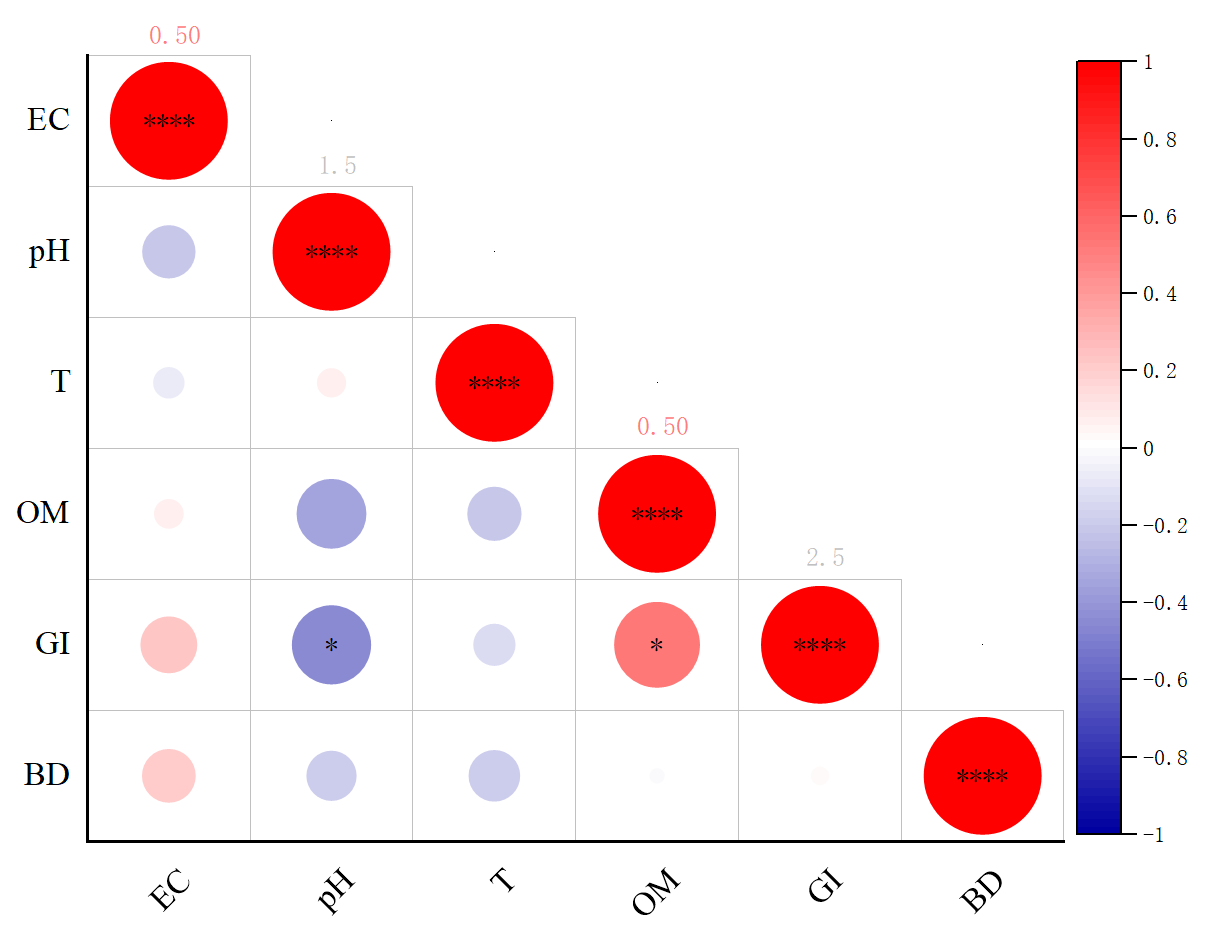


**Figure S2.** Heatmap analysis of abiotic parameters (pH, EC, OM, T, GI, BD) in compost amended with different nitrogen sources (CM: chicken manure; SP: soy powder; FM: fish meal)


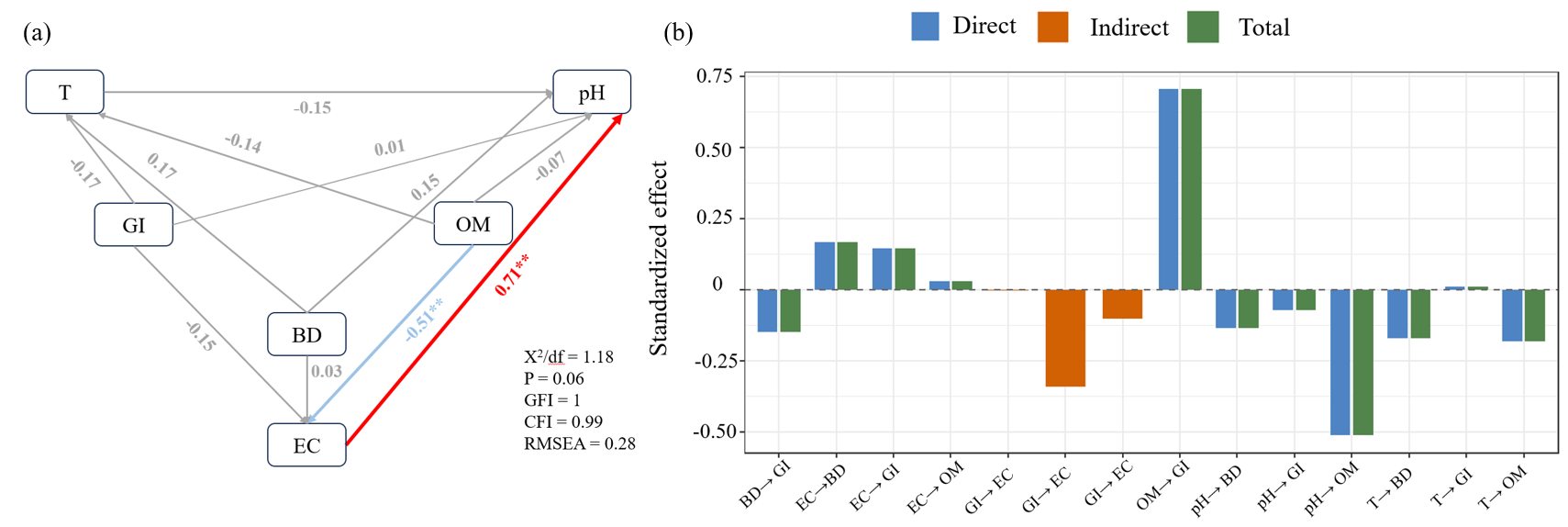


**Figure S3.** Structural equation modeling of compost parameters affecting seed germination: (a) Path diagram of direct effects, and (b) Decomposition of standardized effects.


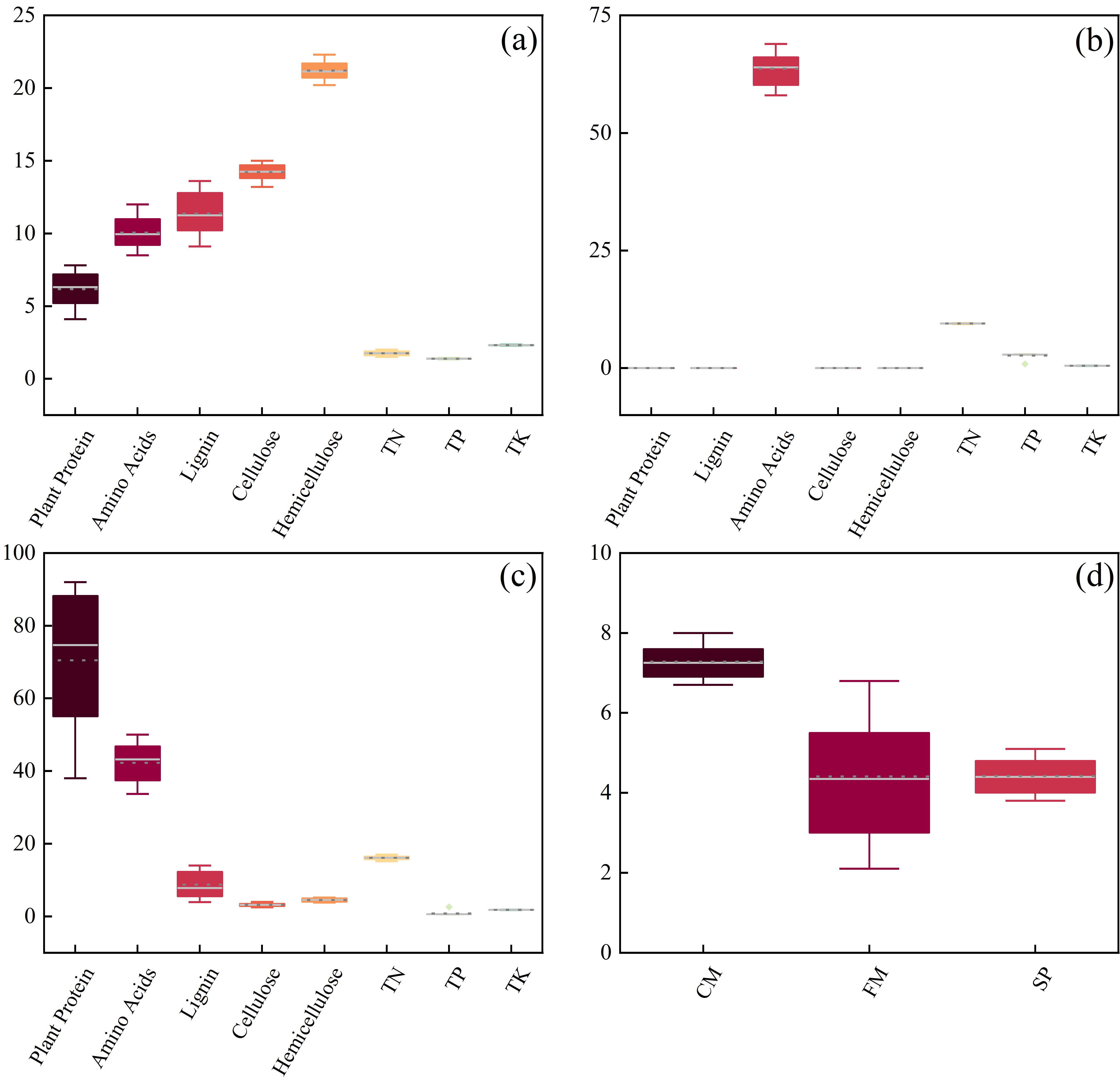


**Figure S4.** Boxplots of Nutrient Composition and pH in Composting Treatments:(a-c) Percentage content of plant protein, amino acids, lignin, cellulose, hemicellulose, total nitrogen (TN), total phosphorus (TP), and total potassium (TK) in chicken manure (CM), fish meal (FM), and soybean powder (SP);(d) pH values of CM, FM, and SP treatments.


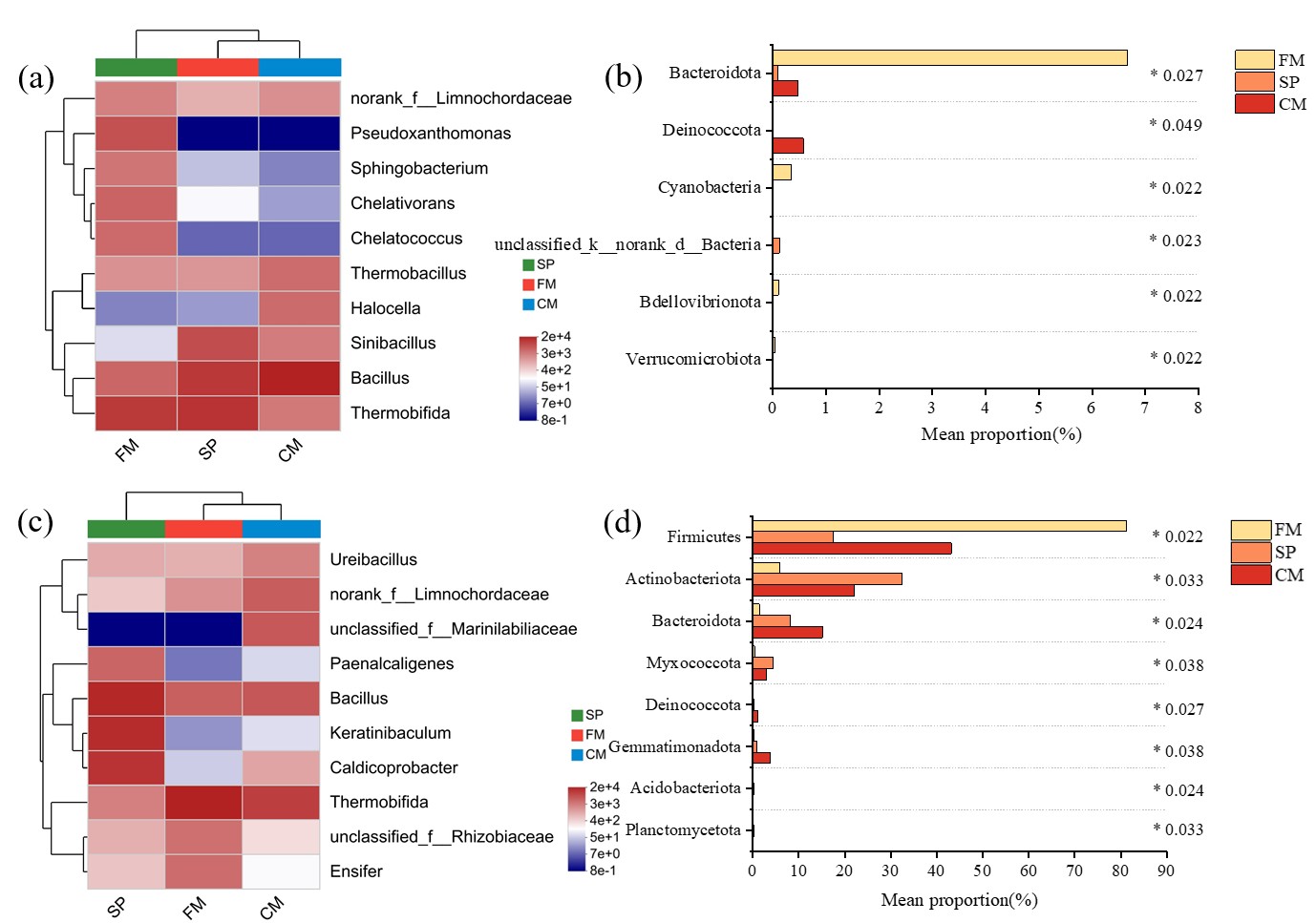


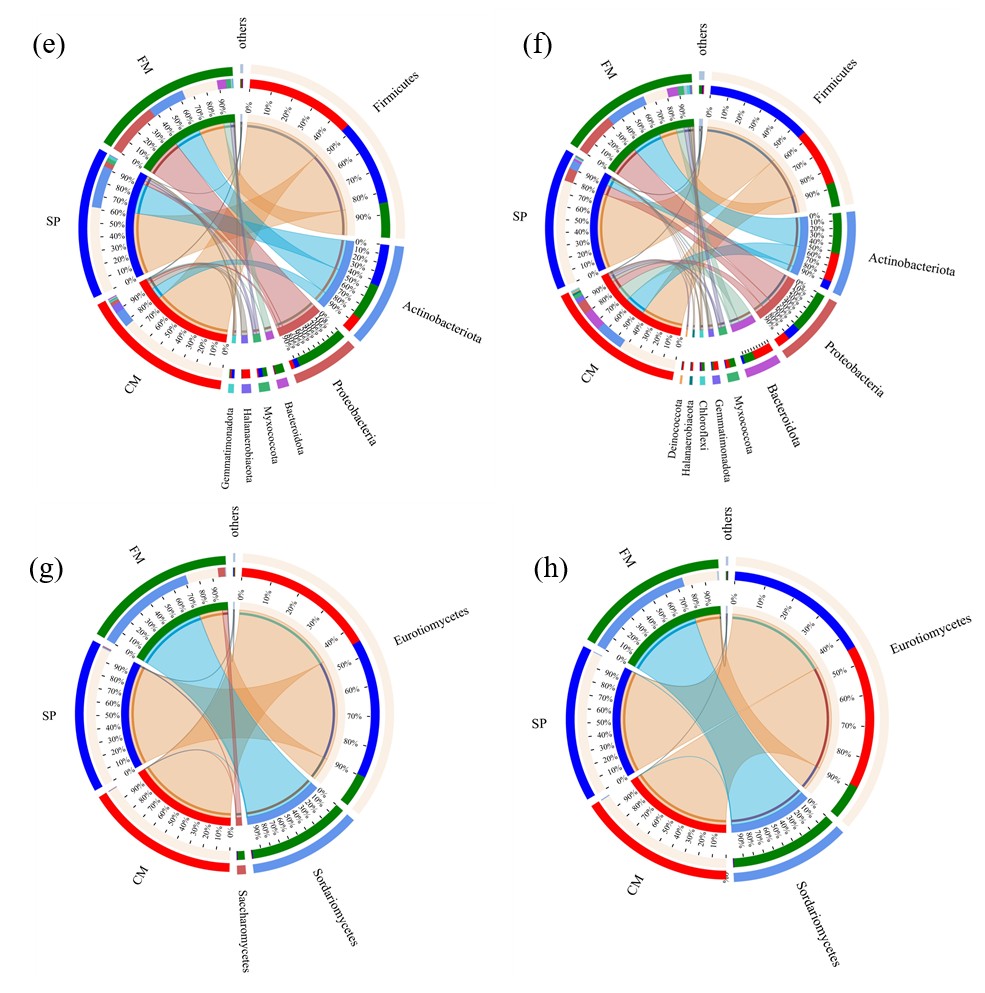


**Figure S5.** Bacterial taxonomic differences and sample-taxon associations during composting. Heat maps of the top 15 bacterial genera on day 12 (a) and day 20 (c). Significance test of bacterial communities at the phylum level on day 12 (b) and day 20 (d). Circos plots of bacterial communities on day 12 (e) and day 20 (f), and fungal communities on day 12 (g) and day 20 (h). CM, chicken manure; SP, soy powder; FM, fish meal.

Table S1 Composition of raw materials for different composting treatments.

| Treatment | Chicken manure  (%, dw) | Soybean powder  (%, dw) | Fish meal  (%, dw) | Straw  (%, dw) |
| --- | --- | --- | --- | --- |
| CK | 0 | 0 | 0 | 100 |
| CM | 20 | 0 | 0 | 80 |
| SP | 0 | 20 | 0 | 80 |
| FM | 0 | 0 | 15 | 85 |

Table S2 Physical and chemical parameters of composting materials.

|  | Straw | Urea | CM | SP | FM |
| --- | --- | --- | --- | --- | --- |
| pH | 6.74 | 8.54 | 7.94 | 6.22 | 6.04 |
| EC (ms/cm) | 5.39 | 0.08 | 5.43 | 3.05 | 9.11 |
| TN (g/kg) | 6 | 460 | 34 | 50 | 80 |
| TC (g/kg) | 420 | 0 | 450 | 400 | 350 |
| C/N | 70 | - | 13.2 | 8.0 | 4.4 |
| MC (%) | 15 | 0 | 41.5 | 10 | 2 |

Note: MC, moisture content.

Table S3 Original physichemical parameters of composting treatments.

|  | CK | CM | SP | FM |
| --- | --- | --- | --- | --- |
| pH | 6.74 | 7.94 | 6.22 | 6.04 |
| EC (ms/cm) | 5.39 | 5.43 | 3.05 | 9.11 |
| TN (g/kg) | 6 | 11.6 | 14.8 | 17.1 |
| TC (g/kg) | 420 | 426 | 416 | 409.5 |
| C/N | 70 | 58.64 | 57.6 | 60.16 |
| MC (%) | 15 | 20.3 | 14 | 13.05 |

Note: MC, moisture content.
